# Use of cracked maize as a carrier for NDV_4 _vaccine in experimental vaccination of chickens

**DOI:** 10.1186/1743-422X-7-67

**Published:** 2010-03-23

**Authors:** Atanda O Olabode, James A Ndako, Georgebest ON Echeonwu, Obinna O Nwankiti, Anthony A Chukwuedo

**Affiliations:** 1 Department of Virology, Federal College of Veterinary and Medical laboratory Technology, Vom, Nigeria; 2 Poultry Viral Vaccine production Department, National Veterinary Research Institute, Vom Nigeria; 3 Rabies Diagnostic section, National Veterinary Research Institute, Vom, Nigeria

## Abstract

The suitability of V_4 _vaccine coated on cracked local grain (maize) and its husks and used for oral vaccination of chickens was assessed. Seventy-two (72) birds aged three (3) weeks and above were divided into six groups of twelve (12) birds per group. The birds were bled to determine their prevaccination HI antibody status while five different samples of cracked maize were coated with the V_4 _vaccine and fed to the chickens orally in each of the groups. All birds in the group including the controls were bled at 7, 14 and 21 days post vaccination to determine the presence and level of antibody response in each of the groups. Results obtained showed that prevaccination haemagglutination inhibition (HI) titre was less than two (log_2_) in 18% of the birds used in this experiment, however 14% of the birds had an HI titre of ≤ 4. The post vaccination antibody titre showed that birds vaccinated with vaccine coated maize gave a post vaccination HI antibody titre of between Log_2_(6-8). when the coated maize samples were soaked in water at room temperature and assessed after 24 hours, the treated maize parts gave >6.3 log_10 _EID_50 _and above while the untreated parts gave < 3.0 log_10 _EID_50_. The experiment showed that whole maize and husks, which were not treated, may contain agents which are virus inhibitory. Form this research the treated maize which was soaked and washed gave a higher geometric mean titre, hence tends to be good carriers of the virus (vaccine). It is therefore concluded from this work that processed cracked maize could be a good carrier of NDV_4 _vaccine. It is hereby recommended that only treated maize could be used as carrier for the V_4 _vaccine.

## Background

The Newcastle disease virus (NDV) is classified within the genus Paramyxovirus of the family Paramyxoviridae [1]. The virus has a single stranded RNA like other members of the Paramyxovirididae family. The Newcastle disease virus possesses two surface proteins that are important in the identification and biological characteristics of the virus [2].

The disease is primarily a viral disease of chickens in particular and other avian species [3,4]. Human infections has however been reported among laboratory workers and other poultry workers. The disease is characterized by conjunctivitis, without cornea involvement in man, [5].

Newcastle disease is worldwide in distribution [6]. Nevertheless international recording and reporting of Newcastle disease has been carried out by the Food and Agricultural Organization of the United Nations and OIE which form the basis of several assessment of the geographica1 distribution of the disease [6].

The disease has no treatment (i.e. cure) but is however controlled by vaccination using the imported and the three vaccines currently produced at the Virology Division of the National Veterinary Research Institute Vom. It has been established that the Newcastle disease vaccine prevents possible outbreak of the virus [7]. Chickens can be immunized against New castle disease, while low virulence live-virus vaccines are administered by a variety of routes such as drinking water, intra-ocular, intra nasal or by sprays while killed oil emulsion vaccines are administered to pullets intramuscularly or subcutaneously as 'final vaccine prior to the onset of egg production, [8].

All strains of the Newcastle disease virus will agglutinate chicken red blood cells in vitro (and some times red blood cells from other animal species). This biological activity is known as haemagglutination (HA) and is the basis of the common tests to detect haemagglutination viruses in the family. Haemagglutination inhibition (HI) test is used to detect antibodies to this virus, though other serological tests are available, [9,10].

Transmission of the virus is most common through bird to bird and humans or formites usually via droplets from the respiratory tract or through faeces. The virus is shed from infected birds in all secretions and excretions. In dry conditions aerosol borne infected particles promote the spread. Infection is acquired by birds through inhalation of infected droplets or particles or through ingestion of infected food, [11].

In this study therefore, efforts were made to determine the suitability of maize coated with V_4 _virus as a carrier in the vaccination of chickens against Newcastle disease.

## Materials and methods

### Samples collection

One hundred and twenty (120) day old cockerels were used for this research. These birds were obtained and brooded for three weeks, until the maternal antibodies were not detectable according to the method of Allan and Gough [12]. The chickens were fed with chick mash for eight [8] weeks, which were later changed to growers mash at the 9^th ^week. They were maintained on this feed throughout the period of the experiment also the chickens were given portable water twice daily with good poultry management practices, according to the method of Darminto, [13]. Blood samples were collected from the birds at 7 days interval for 21 days. The blood samples were allowed to clot at room temperature, stored at +4°C and centrifuged at 3000 rpm for 5 minutes in a refrigerated centrifuge. The sera collected were then stored at -20°C until ready for use.

### Processing of the grains

Ten measures of maize grain were cracked to small sizes, to ease swallowing by the birds. This was then divided into five equal groups which were fed to the birds in five different cages:

The first group involves the maize husk measuring 72 g this was soaked in water for three days, with daily changes of water throughout the period, after which it was sun-dried, this is to be used for the birds in cage 1.

The second group was also made up of maize husks measuring 72 g which were left unsoaked in water; this is to be used for the birds in cage 2.

The third group was made up of the cracked maize (with removed husks), which was soaked in water for three days, with a daily change of water (1:3; w/v). After the third day, it was sun-dried on a clean and well washed surface this was for the birds in cage 3.

The fourth group included part of the cracked portion above. This was left unsoaked and used for the birds in cage 4. These procedures were according to the method described by Alders, and Spradbrow, [9,10].

The fifth group was made up of maize offal which was obtained by soaking the entire maize grain in water for four days after which it was ground and sieved. The sieved out part (offal) was then sun dried. This was used for the birds in cage 5. This method was adopted by, [9,10].

### Vaccine administration

One ampoule of already freeze dried NDV_4 _vaccine was reconstituted in 5 mls of clean sterile non-chlorinated water. The 5 mls vaccine suspension was further diluted in 50 mls of clean non-chlorinated water and maintained in ice trough, ready for use, According to the method described in the OIE Manual [14].

### Preparation and coating of carrier maize grain with the virus

Forty-eight grams(48 g) of each of these maize carriers were mixed thoroughly and separately with 10 mls of the NDV_4 _vaccine suspension to ensure that the vaccine virus adhered well to the carriers, (this was aseptically done manually, using sterile equipments and hand gloves, while the environment was thoroughly disinfected). They were kept at +4°C ready for use.

### Method of vaccination

The first vaccination of birds was carried out on the 9th week of age; the various groups of birds were vaccinated using the different vaccine carriers. All the birds were starved overnight of feed and water. The vaccines carriers were given as follows:

#### Group A

The birds in group A were fed with the unsoaked maize husk mixed with the V_4 _vaccine.

#### Group B

The in birds group B were given the soaked maize husk carrying the V_4 _vaccine.

#### Group C

The birds in group C were fed with the soaked cracked maize mixed with the V_4 _vaccine.

#### Group D

The birds groups D were fed with the unsoaked cracked maize combined with the V_4_ vaccine.

#### Group E

The birds in group E were vaccinated using the maize offal mixed with the V_4 _vaccine.

#### Group F

The group F birds were kept as control birds without any vaccination (fed with carrier without vaccine coating).

All the birds and the controls were adequately provided with portable water and maintenance feed.

### Booster vaccination

The second vaccination (booster dose) was carried out after 21 days from the first vaccination at the twelfth (12^th^) week of age. This was done using the same vaccine carriers, quantities and procedures as it was done in the first vaccination in all the groups.

### Post vaccination blood sample collection

1 ml each of blood samples were collected from all the experimental birds at day 7, 14 and 21. The bleeding process was carried out aseptically as described earlier and the blood was put into sterile and dry vials and allowed to clot overnight. These were then centrifuged at 3000 rpm for 5 minutes in a refrigerated centrifuge. The 3 sera samples collected per bird were then stored at -20°C until ready for use, according to the method of Alders and Spradbrow [9].

### Haemagglutination test

The presence and titer of the haemaggluting virus were tested using the method of Allan and Gough (1976). A U- bottomed shaped microtitre Perspex plate was used for the virus titration. The antigen (1: 1 0 dilution) was prepared in phosphate buffer saline (PBS, P^H ^7.2). One row of ten wells were marked A1-10 was used for each antigen to be tested. Two other wells A11 and A12 were used for cell and antigen control.

### Haemagglutination inhibition test

The haemagglutination Inhibition (HI) test, was based on the inhibition of viral agglutination by the specific serum antibody, according to the method of Allan and Gough, [15]. A twofold serial dilution in PBS of the Birds (specific) serum was made from the first well to the eleventh well (i.e.) wells 1 - 11. After which 0.025 ml of the virus dilutions containing 4HAU was added to each of the serum dilution. It was uniformly mixed and incubated at room temperature for 45 minutes; this is to enable the virus - serum mixture to react. After this 0.025 ml of 1% suspension of the chicken red blood cell was added to each of the mixtures in the wells and allowed to settle for 60 minutes at + 4°C, while the 12^th ^well contains only PBS and the 1% suspension of the chicken red blood cell. The serum titre were then read, as the highest dilution of serum inhibiting haemagglutination by the virus, which is expressed as the reciprocal of that serum dilution, while the control well shows a clear and visible button of the red cells.

### Eid_50 _determination

One gram of each V_4 _virus coated maize grain samples, were kept into a sterile McCartney bottle and 10.0 ml of PBS containing antibiotics, was ascetically added, shaken and allowed to stand for 24 hours. The mixture was then spun at 3000 rpm for 30 minutes at 4°C in a refrigerated centrifuge. The supernatant were collected and diluted serially in tenfold from 10^-2^-10^-10^. Beginning from the highest dilution, 0.1 ml of each dilution was inoculated into 5 - 10 days old embryonated egg already prepared for inoculation. The inoculated eggs were then sealed with molten candle wax and incubated for 3 days at 37°C along with the control eggs containing no inoculums. At the end of incubation the eggs were recandled to remove the dead eggs, while the viable ones were chilled in the cold room at 4°C overnight. After chilling, haemagglutination spot test was performed on each egg to determine the presence of virus, by mixing a drop of the allantoic fluid with a drop of 10% washed chicken. Red blood cells on a clean white tile mixed and rocked.

Those that showed haemagglutination reaction were considered positive (+) for each dilution and the E1D_50 _was calculated using the Reed and Muench method, [16].

## Results

### Haemagglutination inhibition (HI) titration results

The result obtained from the screened birds showed a mean prevaccination haemagglutination inhibition titre (HI) titer of < 2 which implies that the birds had no detectable antibody to the virus. Details as presented in table 1. When the birds were later vaccinated, the results of HI post vaccination at 7, 14 and 21 days showed a higher HI titer. The results obtained from soaked cracked maize grain, maize offal, and soaked husk of the maize grain samples respectively showed a rise in antibody titer when fed to the birds, as seen on table 2. The result obtained from unsoaked samples of the cracked maize grain and husks respectively observed a drop in titer to the vaccine for birds in that group. The highest HI titer result was obtained from soaked cracked maize carrier followed by soaked maize husk. The maize offal was used as a control, because it is known to have been processed through water and has been reported to have no virucidal effect on the Newcastle disease virus [7]. While the result obtained from the maize offal is as shown in Table 3.

**Table 1:** Prevaccination HI test result

Group	Number of Birds	Detectable Antibody Titer(Log_2_)
A	12	<2
B	12	<2
C	12	<2
D	12	<2
E	12	<2
F	12	<2

6	72	<2

**Table 2:** HI titres of post vaccination for the birds screened.

Sampling periods	HI titer level (Log_2_)			
(Days)	A	B	C	D
7	2	7	7	6
14	2	7	8	7

21	4	9	7	7

**KEY**:

A - Titer obtained from birds fed with unsoaked cracked maize coated with the V_4 _Vaccine.

B - Titer obtained from birds fed with soaked cracked maize coated with the V_4 _Vaccine.

C - Titer obtained from birds Fed with soaked husk mixed with the V_4 _Vaccine.

D - Titer obtained from birds Fed with the Unsoaked maize husk mixed with the V_4 _Vaccine.

**Table 3:** HI means titre of post vaccination for birds fed with maize Offal mixed with the V_4 _Vaccine.

Sampling periods (Days)	N0. of birds	Post Vaccination HI titer
7	12	7
14	12	7
21	12	9

### Statistical analysis

Using two factor analysis of variance, the birds showed significant mean antibody geometric titer (P > 0.05) during the post vaccination period using the differently processed maize grain sample. There seem to be a steady rise in the antibody titre as the number of days of repeat vaccination increases. Applying the Multiple Range Test (MRT), it was observed that the various parts of grain used enhanced considerable intake of the vaccine by the birds and by implication an enhancement of antibody generation.

## Discussion

The ability of maize as a carrier food to deliver viable vaccine virus in order to stimulate the production of protective antibody amongst chickens was the main focus of this work. The V_4 _(vaccine), a thermostable strain of NDV has been used in the control of Newcastle disease in both exotic and local chicken [1-3]. However this study was carried out to identify a suitable maize carrier for ease of vaccinating local and free range chickens.

In this work different processed maize grain components mixed (coated) with V_4 _vaccine suspension was used to vaccinate chickens experimentally, the result obtained showed that the cracked maize that were soaked gave an appreciable high titre compared to those parts that were Unsoaked. This has been attributed to the removal of the effects of some inactivating substances on the grains(such as acids, lectins and other inhibitory substances) by soaking in water for 3 days. This is similar to the work of Olabode [7], who used Offal as carrier for the V_4 _Vaccine in Nigeria.

According to Agbor [17] some grains (e.g. maize, rice and, guineacorn) were all found to contain substances inherent in them capable of inactivating the V_4 _thermostable Newcastle Disease Virus when coated on them. This was evidenced with the reduced concentration of the virus in the unsoaked maize when inoculated into embryonating eggs giving a 4.3 log_10 _EID_50_.

Low titer obtained when the unsoaked maize samples were used as carrier for the V_4 _vaccine, showed that unsoaked grains could not have been a successful delivery vehicle for the V_4 _Vaccine compared with the soaked samples which gave a 6.3 logs EID_50_.

According to Rehmani and Spradbrow [18]; McMillan *et al.*, [19] it was observed that the condition for successful use of any chosen food as vaccine carrier is the ability of such food to allow firm binding or adherence of the coated vaccine virus without interfering with the virus viability. According to Echeonwu *et al. *[20] who used Cassava granules as a carrier for the (NDV) strain V4 UPM on free range chickens, it was also reported that lectins play important roles in such virus binding or adherence to food grain surface, hence the need for soaking such grains in water.

The V_4 _Vaccine coated on the soaked grains were seen to be viable for up to 24 hours at room temperature, with only a minimal loss in titre. Although the V_4 _vaccine is thermostable, the vaccine must be used as soon as possible and with out exposure to direct sunlight.

## Conclusion

This method of vaccination will go a long way to reduce Newcastle Disease related mortalities, thereby improving confidence in village chicken farmers hence contributing to poverty alleviation in the rural areas, more so the method does not require direct contact with the birds during vaccination thereby reducing stress associated with handling birds for individual vaccination coupled with ease of application by poultry farmers; vaccine delivery through this medium to chickens of all ages has been able to give a good result, hence might prove better than other conventional methods.

## Competing interests

The authors declare that they have no competing interests.

## Authors' contributions

AO conceived of the study and carried out the study design. JA drafted the manuscript, carried out the various analyses, including data collection and statistical analysis. GE participated in the study coordination. ON participated in data collection. AC contributed in data analysis. All authors read and approved the final manuscript.
